# Characterization of muscle synergy similarity and adaptation in hip exoskeleton-assisted locomotion

**DOI:** 10.3389/fbioe.2025.1679101

**Published:** 2025-09-19

**Authors:** Ruiming Wang, Dewei Liu, Linfan Yu, Xiaoguang Liu, Haoyu Wang, Wei Yang

**Affiliations:** ^1^ Feinberg School of Medicine, Northwestern University, Chicago, IL, United States; ^2^ Ningbo Global Innovation Center, Zhejiang University, Ningbo, China; ^3^ Hospital Department, Ningbo Rehabilitation Hospital, Ningbo, China

**Keywords:** wearable device, hip exoskeleton, walking assistance, surface electromyography, muscle synergy

## Abstract

Recent advancements in robotic exoskeleton technology have demonstrated significant potential in reducing users’ energy consumption and muscle activation. However, how users adjust muscle recruitment and coordination in expenditure and muscle activation levels. However, the mechanisms underlying users’ adaptation of muscle recruitment and coordination patterns in response to external robotic assistance remain poorly understood. This study introduces a novel methodological framework for quantifying the impact of assistance on human muscle synergy patterns through similarity analysis, which incorporates a weighted summation of Pearson correlation coefficients between paired synergies. Eight healthy adult participats underwent treadmill walking trials under two conditions: with and without a portable hip exoskeleton. The experimental protocol consisted of two distinct sessions. In the first session, participants walked with varying assistive torque, enabling comparative analysis of muscle synergies across different conditions. The second session, involved a temporal adaptation assessment, where participants initially walked in a 2-min zero torque (ZT) mode, followed by a 10-min assistive mode, and concluded with another 2-min ZT mode. The analysis revealed that four primary synergies accounted for 92.73% ± 0.43% and 93.06% ± 0.64% of the variance in surface electromyography (sEMG) signals during exoskeleton-assisted and unassisted walking, respectively. The developed similarity indices proved effective in quantifying significance differences in muscle synergy patterns between assisted and unassisted conditions. These findings provide valuable insights into neuromuscular control mechanisms during exoskeleton-assisted locomotion, contributing to the optimization of robotic assistance strategies.

## 1 Introduction

Over the past few decades, powered exoskeleton technology has demonstrated significant potential in enhancing human locomotion performance (Sawicki et al., 2020; C. Yang et al., 2022; W. Yang et al., 2021) and facilitating motion rehabilitation (X. Li et al., 2017; Wei et al., 2020; W. Yang et al., 2020). Recent advancements in exoskeleton and exosuit control systems for gait assistance have shown substantial reductions in both energy expenditure and muscle activation levels (Gordon et al., 2022; Ishmael et al., 2021). Various optimization parameters have been extensively studied, including human-robot interaction forces (Kim et al., 2019; Lee et al., 2021; J. Zhang et al., 2017), user preferences (Ingraham et al., 2022), robot admittance (T. Zhang et al., 2019), and transferred work (Tu et al., 2021), the impact of exoskeletal assistance on users’ muscle recruitment and coordination patterns remains underexplored. A comprehensive understanding of neuromuscular control adaptations in response to external assistance (Steele et al., 2017) or unexpected perturbations (Shan et al., 2025) is crucial for developing advanced control strategies in wearable robotics.

The concept of muscle synergy, first proposed by Bernstein (1967), provides a theoretical framework for understanding the organization of motor control in the central nervous system (CNS). This hypothesis suggests that the CNS coordinates movement through the combination of low-dimensional muscle group patterns, thereby reducing the complexity of musculoskeletal control (d’Avella et al., 2003). Extensive research in both animal models and human subjects has validated the utility of muscle synergy analysis in characterizing neuromuscular coordination patterns (Chvatal et al., 2011; d’Avella and Lacquaniti, 2013; Muceli et al., 2010; Saltiel et al., 2001; Torres-Oviedo et al., 2006). Subsequent studies have investigated muscle synergy characteristics across various motor tasks, including gait (Chvatal and Ting, 2013; Hagedoorn et al., 2022; Zych et al., 2021), running (Bach et al., 2021), turning (Choi et al., 2019), and cycling (Barroso et al., 2014). Notably, Saito et al. (2021) demonstrated that 13 distinct muscle synergies could account for the majority of motor tasks through different combinations of these patterns.

The application of muscle synergy analysis has extended to populations, providing insights into neuromuscular adaptations following injury or disease (Chen et al., 2021; Goudriaan et al., 2022; Mizuta et al., 2022; Serrancolí et al., 2016; Shuman et al., 2018). In the field of exoskeleton research, synergy analysis has emerged as a valuable tool for evaluating device-user interactions. For instance, Z. Li et al. (2019) quantitatively assessed lower limb muscle synergies during hip-knee exoskeleton-assisted gait, revealing significant alterations in synergy patterns. Liu and Gutierrez-Farewik (2023) demonstrated the utility of muscle synergy analysis identifying locomotion mode transitions, potentially informing exoskeleton control strategies. Steele et al. (2017) further validated the application of exoskeleton in rehabilitation through comparative analysis of synergy patterns under different torque conditions from an ankle exoskeleton.

Despite these advancements, current research primarily focuses on comparing muscle synergy patterns between specific exoskeleton assistance modes and unassisted conditions. The effects of varying assistance levels and the temporal dynamics of neuromuscular adaptation remain poorly understood. Given the complex musculature surrounding the hip joint compared to other lower limb joints, this study employs a portable hip exoskeleton during treadmill walking to investigate muscle synergy patterns under varying assistive torque conditions and during assistance mode transitions.

Our goal was to systematically investigate the influence of external robotic assistance on human muscle synergy patterns. The present study makes two significant to the field: (1) the development of a novel synergy similarity index for quantifying differences between assisted and unassisted muscle synergy patterns, and (2) the investigation of neuromuscular adaptations during continuous assistance mode transitions. To our knowledge, this represents the first systematic investigation of muscle synergy patterns during portable hip exoskeleton-assisted gait. These findings valuable insights for optimizing exoskeleton control strategies and advancing our understanding of neuromuscular control mechanisms in assisted locomotion.

## 2 Methods

### 2.1 Exoskeleton system

**FIGURE 1:**
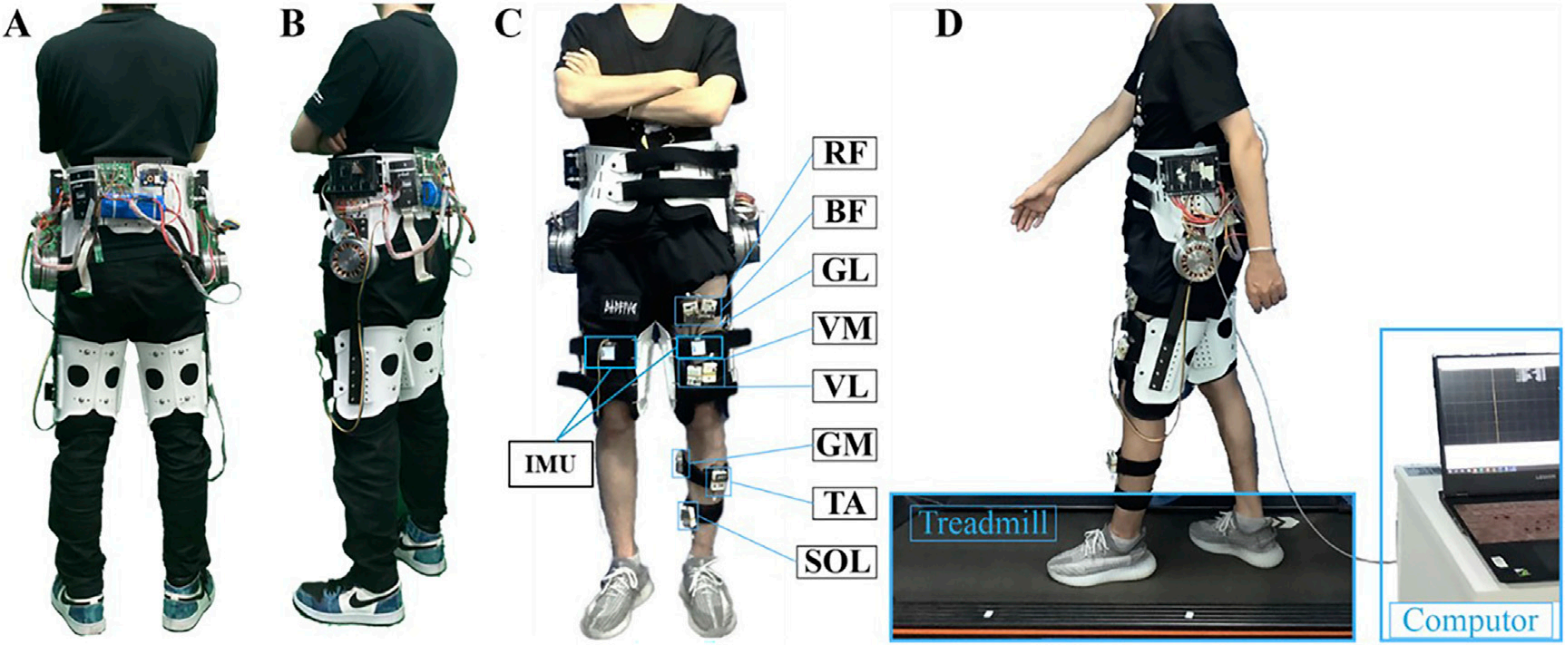
System overview. **(A)** Rearview of the hip exoskeleton prototype. **(B)** Sideview of the hip exoskeleton prototype. **(C)** One subject wearing the hip exoskeleton with eight sEMG sensors on the left thigh and shank. **(D)** Treadmill walking test with a computer collecting the IMU and EMG signals.

The bilateral hip exoskeleton employed in this study represents an enhanced iteration of the original prototype developed by Xu et al. (2023). The system architecture comprises six primary modules: (1) mechanical structure, (2) bandage interface, (3) control unit, (4) motor joint assembly, (5) motion perception system, and (6) power supply module, as illustrated in Figure 1. The hip exoskeleton has a total mass of 4.95 kg and delivers a rated torque of 23 Nm for both hip flexion and extension movements.

The robotic hip joint mechanism integrates three key components: a servo motor (Maxon EC 90 Flat, Maxon motor Co., Ltd., Sachseln, Switzerland), a custom-designed planetary gear reducer, and a motor driver (ESCON 50/5 module, Maxon motor Co., Ltd., Sachseln, Switzerland). The motion perception module system incorporates two inertial measurement units (IMUs, JY901, Wit-Motion, Inc., Shenzhen, China) mounted on the anterior aspect of each thigh segment. The entire system is powered by a 24V lithium-ion battery pack and controlled yhrough a dedicated microcontroller unit.

### 2.2 Control architecture

The whole control framework of the hip exoskeleton could be divided into two levels, the high-level control and the low-level control. High-level control is used to identify the human walking state and generate desired torque profile. The low-level control aims to ensure the desired torque is accurately tracked.

**FIGURE 2:**
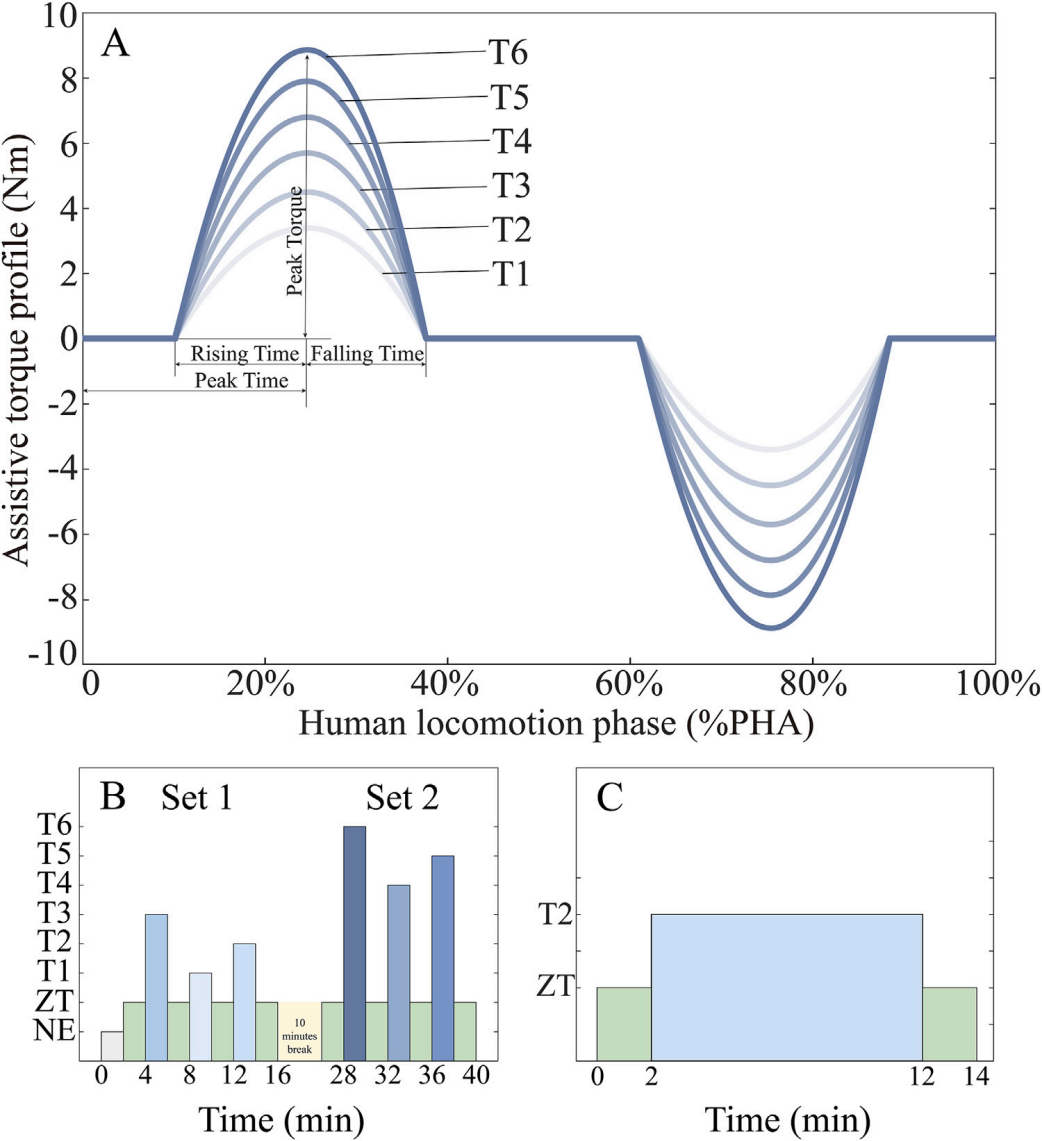
Experiment protocol. **(A)** The assistance profile is a centro-symmetric curve defined by rising time, falling time, peak time, and peak torque of extension and flexion which always start at the heel strike (0% of the gait sycle). In this study, the torque amplitude changes while the assistive time remains unchanged in every gait sycle. **(B)** Variable peak torque section. In the beginning, the NE mode is conducted as the baseline. Two sets are separated by a 10 min break. **(C)** The assistance on-off adaptation section. The assistive torque switches from ZT mode to T2 mode, and ends with ZT during the assistance on-off adaptation session.

In the high-level control, the user’s gait phase is estimated through phase-based oscillator (PO), an efficient and simple method that only relies on the hip joint angle and angular velocity in sagittal plane. According to human walking biomechanics (C. Yang et al., 2022), we defined the assistive torque profile with six feature points (corresponding to four parameters), and curves connected those points smoothly by quadratic functions. Thus the assistive torque profile can be determined by rising time, falling time, peak time, and peak torque of extension and flexion torque (Figure 2A). In our previous work, the optimal assistive torque profile had been defined by means of human-in-the-loop (HIL) optimization (Ma et al., 2024). In this study, only the peak torque is adjusted among the four parameters obtained by HIL optimization to explore the impact of different assistive torque amplitudes on human muscle recruitment and coordination. Six different peak torques are 15%, 20%, 25%, 30%, 35%, and 40% of rated torque, represented as T1, T2, T3, T4, T5, and T6, respectively. According to our pre-tests, too-high peak torque (>0.20 Nm/kg) may lead to unnatural gait, while a too-low peak torque (<0.05 Nm/kg) provides little assistance which is not noticeable to the users.

### 2.3 Testing protocol

The goal of the experiment is to investigate neuromuscular control adaptations under external assistance and to quantify muscle synergy pattern similarity through weight extraction compared to normal walking. The protocol consisted of three distinct sections: (1) familiarization training, variable peak torque assistance assessment, and (2) assistance on-off analysis. Throughout all sections, participants maintained a constant treadmill speed of 3 km/h.

The familiarization training section was conducted 24 h prior to formal testing. During this section, participants completed a minimum of 10 min of treadmill walking while wearing the hip exoskeleton with each assistive torque pattern. This ensured adequate adaptation to the assistance and minimized potential conscious resistance to the applied torque.

The variable peak torque assistance phase commenced with a 2-min baseline trial without exoskeleton (NE) to establish reference measurements. Subsequently, participants performed exoskeleton-assisted walking trials with randomized torque patterns. Each assistance condition was followed by a zero-torque (ZT) mode, implemented through comprehensive compensation model that accounted for friction, inertia, and gravitational forces. This ZT mode served to mitigate potential carryover effects from preceding assistance conditions. Each trial consisted of a 2-min walking period, with the initial minute allocated for adaptation and stabilization, and the final minute reserved for data analysis. The experimental sequence for this section is detailed in Figure 2B.

The assistance on-off transition section, illustrated in Figure 2C, started with a 2-min ZT mode and followed by a 10-min T2 assistive mode (selected based on participant preference for optimal comfort and natural movement). The section concluded with an additional 2-min ZT mode to examine temporal changes in muscle coordination patterns and assess synergy adaptations during transitions between assisted and unassisted walking conditions.

### 2.4 Participants and data collection

Eighthealthy volunteers (one female and seven males; ages: 25.0 ± 3.4 years; weights: 61.5 ± 9.5 kg; heights: 170.3 ± 9.9 cm; mean ± standard deviation) without history of lower limb injuries musculoskeletal disorders participated in this study. All participants provided written informed consent prior toparticipation, and the experimental protocolwas approved by the Ethical Committee of Zhejiang University.

Kinematic data were collected using an IMU mounted on the anterior aspect of the thigh, which recorded hip flexion/extension angles and angular velocities at a sampling frequency of 200 Hz. Gait cycle estimationwas performedusing a phase oscillator algorithm, which processes the IMU-derived angle and angular velocity data to provide real-time gait phase information.

Muscle activity was monitored using wireless surface electromyography (sEMG) sensors (Zhou et al., 2021). Based on analysis of open-source experimental data demonstrating significant bilateral symmetry in muscle synergy patterns (Feng et al., 2024), sEMG signals were collected from eight major muscles of the left lower limb: rectus femoris (RF), vastus medialis (VM), vastus lateralis (VL), biceps femoris (BF), gluteus maximus (GL), gastrocnemius (GM), tibialis anterior (TA), and soleus (SOL). These muscles were selected based on their primary contributions to human locomotion.

The sEMG signals were acquiredat 2 kHz and processed using the following sequence: (1) band-pass filtered (20–300 Hz) with a second-order Butterworth filter, (2) full-wave rectification, and (3) low-pass filtering (10 Hz) with a second-order Butterworth filter. Processed signals were normalized to 100 points per gait cycle, with amplitudes normalization performed relative to the peak activation value within each gait.

### 2.5 Muscle synergy and similarity

**FIGURE 3:**
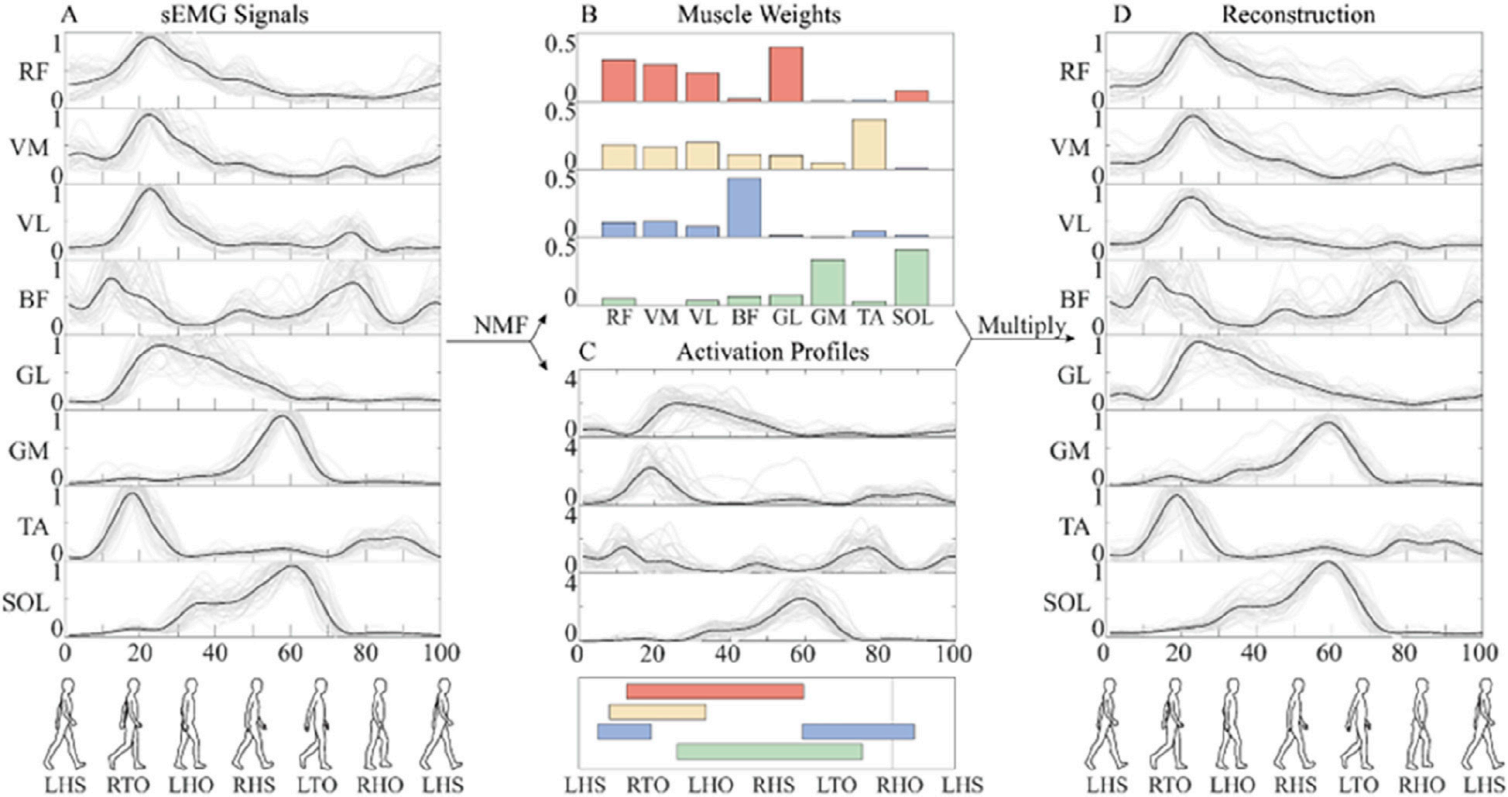
The flow chart of muscle synergy analysis. **(A)** The mean value (black line) of preprocessed sEMG signals of different gaits (gray line). **(B)** Muscle synergy weights. **(C)** Activation coefficient. **(D)** Reconstruction of sEMG signals. In order to facilitate the analysis, the whole gait cycle is divided by left heel strike (LHS), right toe-off (RTO), left heel off (LHO), right heel strike (RHS), left toe-off (LTO), and right heel off (RHO). The activation of four synergies are shown in different colors.

Muscle synergy extraction was performed using non-negative matrix factorization (NMF), a widely adopted method that ensures non-negative features in the decomposed matrices (Hu et al., 2019; Rabbi et al., 2020). This approach provides greater physiological interpretability compared to alternative decomposition methods. We implemented projected gradient method for NMF (Lin, 2007), as shown in Equation 1, which demonstrates superior convergence properties relative to traditional multiplicative update approaches.

$$M_{m\times n}=W_{m\times s}\times H_{s\times n}+E_{m\times n}$$ (1)

where $M_{m\times n}$ represents the preprocessed sEMG dataset (Figure 3A), with *m* denoting the number of muscles (8 channels) and *n* representing the number of the time frames for synergy extraction. $W_{m\times s}$ is the muscle weights matrix (Figure 3B), quantifying the relative contribution of each muscle to the identified synergies. $H_{s\times n}$ is the activation profile matrix (Figure 3C), describing the temporal activation patterns of each synergy throughout the gait cycle. $E_{m\times n}$ represents the reconstruction error between the original and the original sEMG signals (Figure 3D). The optimal number of synergies (*s*) was determined using the variability accounted for (*VAF*) criterion (Chia Bejarano et al., 2017), calculated as Equation 2:

$$VAF=1-\frac{\sum_{i=1}^{m}\sum_{j=1}^{n}{E_{i,j}}^{2}}{\sum_{i=1}^{m}\sum_{j=1}^{n}{M_{i,j}}^{2}}$$ (2)

Consistent with established protocols (Clark et al., 2010), the number of muscle synergies was selected based on two criteria: (1) total *VAF* exceeding 90%, and (2) *VAF* improvement of less than 5% when adding an additional synergy.

To quantify muscle synergy similarity across conditions, we developed an evaluation index η . Due to the random initialization of the NMF algorithm, the order of the column vectors in *W* (muscle synergy weights) and row vectors in *H* (activation coefficients) requires systematic alignment. For comparative analysis, vectors were rearranged according to the activation ratio $c_{k}$ (*k* = 1 … *s*) of each synergy, defined as Equations 3, 4:

$$R_{m\times n}^{k}=W_{m\times 1}^{k}\times H_{1\times n}^{k}$$ (3)

$$c_{k}=\left(1-\frac{\sum_{i=1}^{m}\sum_{j=1}^{n}{\left(M_{i,j}-R_{i,j}^{k}\right)}^{2}}{\sum_{i=1}^{m}\sum_{j=1}^{n}{M_{i,j}}^{2}}\right)\times 100\%$$ (4)

where $R_{m\times n}^{k}$ represents the reconstruction matrix for the *kth* synergy, calculated as the product of the *kth* column vector of W and the *k*th row vector of H . The evaluation index η incorporates both the activation ratio and Pearson correlation coefficient $r_{k}$ between synergy weight matrices ${W1}_{m\times s}$ and ${W2}_{m\times s}$ , where ${W2}_{m\times s}$ serves as the reference synergy weight matrix. Prior to correlation analysis, we verified the normality of the synergy weights for each participant and condition through histogram analysis with normal distribution fits and Q-Q plots. Column vectors were pairwise matched to maximize the sum of Pearson correlation coefficients. The evaluation index is defined as Equations 5, 6:

$$\eta =\frac{\sum_{k=1}^{s}c_{k}r_{k}}{\sum_{k=1}^{s}c_{k}}$$ (5)

$$r_{k}=\frac{Cov\left(W1_{m\times k},{W2}_{m\times k}\right)}{\sigma_{W1_{m\times k}}\sigma_{W_{2_{m\times k}}}}$$ (6)

where $Cov\left(W1_{m\times k},{W2}_{m\times k}\right)$ denotes covariance of muscle synergy weight matrices ${W1}_{m\times k}$ and ${W2}_{m\times k}$ , while $\sigma_{W1_{m\times k}}$ and $\sigma_{W2_{m\times k}}$ are standard deviations of ${W1}_{m\times k}$ and ${W2}_{m\times k}$ , respectively.

Furthermore, we computed an additional similarity index α (Steele et al., 2017) for comparative analysis, defined as Equation 7:

$$\alpha =\left(1-\frac{\sum_{i=1}^{m}\sum_{j=1}^{n}{\left(M_{i,j}-W2_{m\times s}\times H1_{s\times n}\right)}^{2}}{\sum_{i=1}^{m}\sum_{j=1}^{n}{M_{i,j}}^{2}}\right)\times 100\%$$ (7)

where $W2_{m\times s}$ is the mean muscle synergy weights during unassisted walking and $H1_{s\times n}$ is the muscle activations while walking with the exoskeleton in different conditions.

### 2.6 Statistical analysis

Statistical analyses were performed using SPSS 18.0 (IBM Corp., Armonk, NY, USA). A non-parametric test was used to check the difference of similarities of muscle synergies in different conditions. One-way analysis of variance (ANOVA) was applied to evaluate the intra-group and inter-group differences across different subjects or conditions. Results were considered significant for *p* < 0.05.

## 3 Results

### 3.1 Muscle synergy weights

**FIGURE 4:**
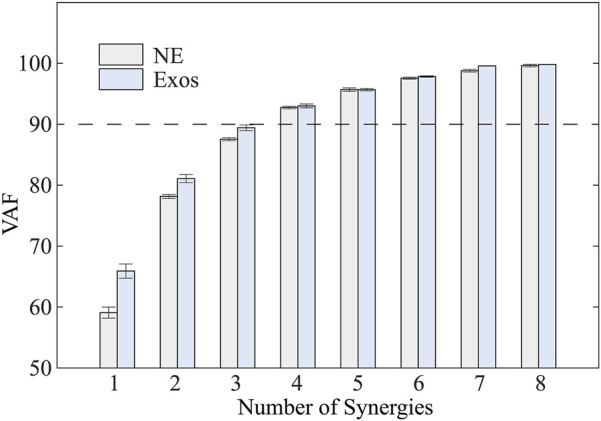
Total variability accounted for (VAF) versus the number of synergies based on NMF. NE and Exos stand for walking trials without and with exoskeleton, respectively. VAF gradually increased with an increase in the number of synergies.

Muscle synergy analysis revealed that four distinct synergies accounted for 92.73% ± 0.43% and 93.06% ± 0.64% (mean ± standard deviation) of the total variance in sEMG signals during walking with and without the exoskeleton, respectively (Figure 4). Although the *VAF* was slightly lower in unassisted walking compared to exoskeleton-assisted conditions, this difference did not influence the optimal number of synergies identified through the *VAF* criterion.

**FIGURE 5:**
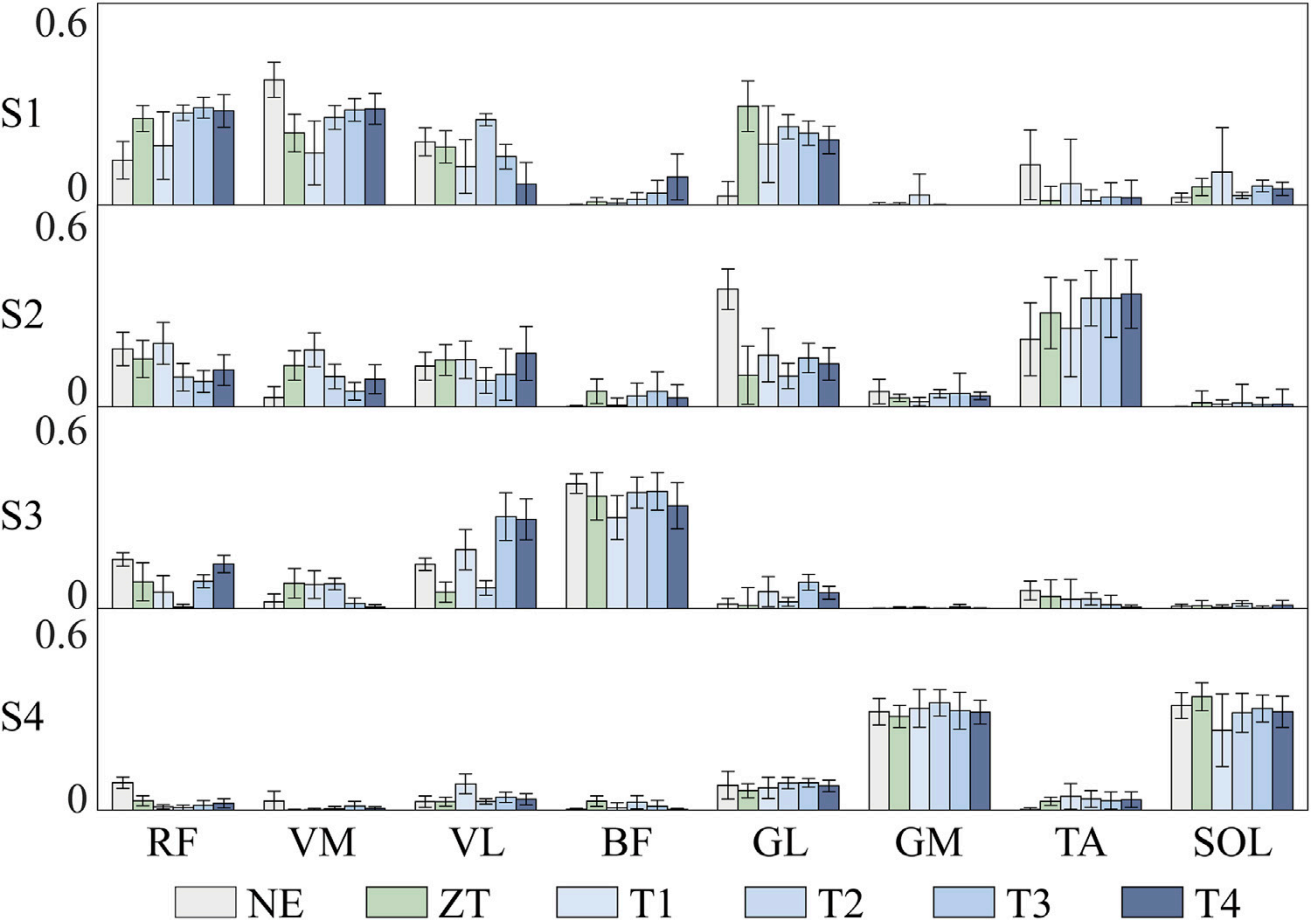
The variable peak torque synergy weight results for Subject one during testing with different exoskeleton assistive modes. Furthermore, the overall synergy weights among different subjects follow a similar trend.

Duringtreadmill walking at a constant speed, participants demonstrated consistent muscle synergy patterns across different assistive torque conditions (Figure 5). The identified synergies exhibited distinct temporal and spatial characteristics: Synergy 1, predominantly involving RF, VM, and VL, was primarily activated during the stance phase. This synergy was associated with weight acceptance following left heel strike (LHS). Synergy 2, characterized by the activation of GL and TA, was initiated during the early stance phase of the left leg. This pattern corresponded to the body weight shift immediately after LHS. Synergy 3, dominated by BF activation, exhibited biphasic characteristics. It was activated during the swing phase to facilitate shank elevation and at the onset of the stance phase to support upright posture. Synergy 4, primarily comprising GM and SOL activation, demonstrated progressive activation throughout the stance phase. The activation level increased gradually, reaching its maximum value during the late stance phase.

### 3.2 Synergy similarity

**FIGURE 6:**
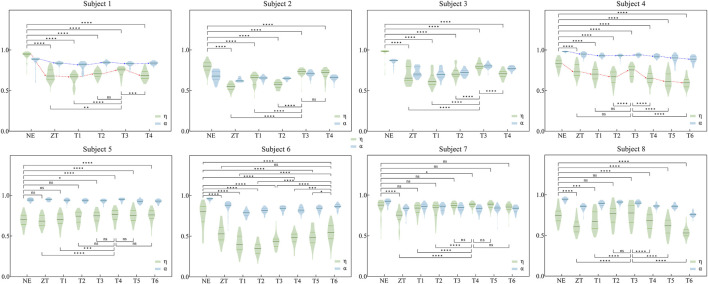
Similarity indices η and α for eight participants under various assistance modes. Comparison conducted between exoskeleton-wearing and non-wearing conditions, as well as between optimal similarity assistance mode and other modes for η (The specific values and analysis of η and α can be found in Supplementary Tables SA1, A2 in Appendix). Significance levels represented by asterisks: ns (p ≥ 0.05), * (p < 0.05), ** (p < 0.01), *** (p < 0.001), **** (p < 0.0001).

The similarity of muscle synergies between exoskeleton-assistedconditions and normal walking was quantitatively assessed using two indices ( α and η ), as illustrated in Figure 6. Statistical analysis revealed significant reductions in both similarity indices during exoskeleton-assisted walking compared to normal walking, even in the ZT condition. Meanwhile, both indices demonstrated a characteristic pattern: initial decrease with increasing assistive torque, followed by subsequent increase, enabling identification of individual-specific optimal assistive torque levels. For η , subjects one and four exhibited maximum median values of 0.7548 and 0.7555, respectively, in T3 condition (p < 0.001).

In contrast, the α index showed less pronounced differences, with subject one reaching a maximum median of 0.8457 in T2 condition (p = 0.9821) and subject four achieving 0.9807 in T3 condition (p = 0.0096). This pattern was consistently observed across all participants.

Detailed quantitative analysis is presented in Supplementary Tables SA1, A2. Table A1 summarizes the median values of both similarity indices across different assistive conditions, with maximum values highlighted in bold. Supplementary Table SA2 statistical comparisons between conditions, where T_MS_ represents the condition corresponds to the maximum median value from Supplementary Table SA1. Comparative analysis revealed that the η index demonstrated more pronounced significant differences in both exoskeleton v.s normal walking comparisons suggesting its superior sensitivity in quantifying the impact of assistive torque variations on muscle synergy patterns during exoskeleton-assisted walking.

### 3.3 Synergy adaption

**FIGURE 7:**
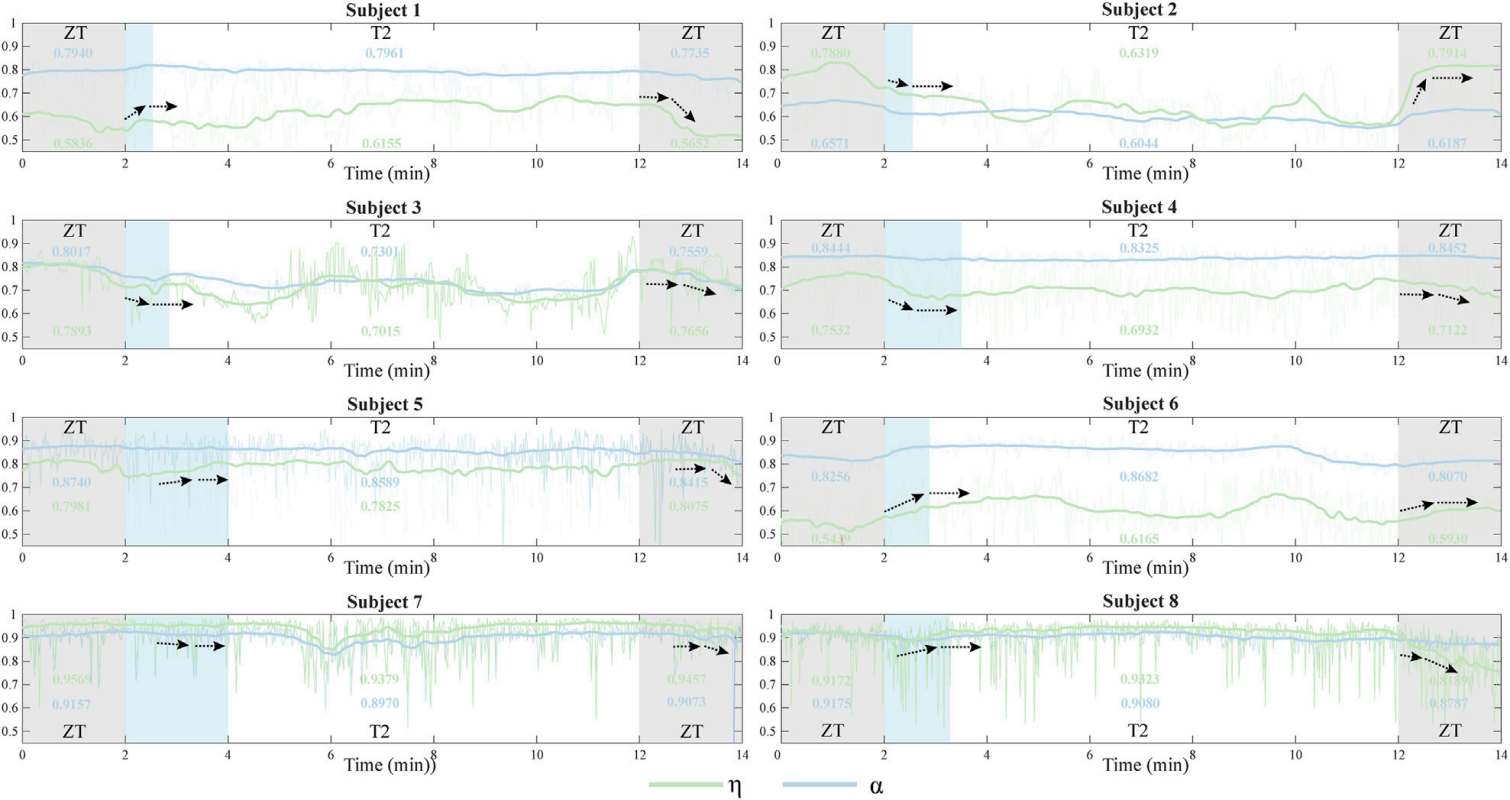
The similarity indices *η* and *α* for eight participants during the entire adaptation phase for assistance switch were categorized into different segments: ZT mode (0–2 min and 12–14 min) and T2 mode (2–12 min). The light blue segments illustrate the process of synergy adaptation during assistance torque transitions. The bold curve represents the average of the original similarity values (light-colored curve) and illustrates the complete synergy adaptation process. Black arrows indicate the process of synergy adaptation during assistance torque transitions.

The temporal adaptation of muscle synergies to abrupt changes in exoskeleton assistance was systematically investigated (Figure 7). The adaptation profiles were characterized using two similarity indices, *η* and *α*, where discrete values were calculated for each gait cycle and subsequently fitted with smoothing curves to illustrate temporal trends. To address the inherent variability in muscle synergy analysis, particularly due to the low Signal to Noise Ratio (SNR) (Grimmer et al., 2022), raw similarity indices (depicted as light-colored lines) were processed using a mean filter to reduce stochastic fluctuations. Analysis revealed that abrupt changes in exoskeleton assistive torque elicited immediate variations in synergy similarity across participants. However, all subjects demonstrated rapid adaptation, achieving a stable state within 2 min (indicated by black arrows). This observed adaptation duration was consistentwith participants’ subjective reports of exoskeleton acclimatization time.

## 4 Discussion

### 4.1 Muscle synergy weights

The analysis revealed that four synergies sufficiently explained the variance in sEMG data during both exoskeleton-assisted and normal walking conditions. This finding aligns with previous NMF-based studies by Zhu et al. (2021) and Cherni et al. (2021), supporting the consistency of dimensionality across different walking conditions. While a general similarity in muscle synergy patterns was observed among participants (Figure 5), consistent with the findings of Z. Li et al. (2019), notable inter-individual variations in muscle-specific contributions within each synergy were evident across different assistive torque levels.

Interestingly, higher assistive torque amplitudes tended to produce muscle synergy more similar to normal walking, though this trend was not universally observed across all synergies. Consequently, to gain a more profound understanding of the similarity between regular walking and exoskeleton-assisted walking under distinct assistive torque conditions, the necessity for a quantifiable similarity index becomes evident.

### 4.2 Synergy similarity

The comparative analysis of similarity indices revealed that the weighted sum of Pearson correlation coefficients *η* provides superior quantification of muscle synergy pattern similarity compared to the conventional index *α*. This superiority manifests in two key aspects: First, η demonstrates greater sensitivity to variations across different assistive torque levels (Figure 6), enabling more precise identification of the optimal assistance modes for individual users. For instance, based on η , participants one and four could unambiguously identify T3 as their optimal assistance mode, whereas α yielded inconclusive results. Second, statistical comparisons (Supplementary Tables SA1, A2) showed that η consistently produced more significant differences (p < 0.05) than α in both exoskeleton versus non-exoskeleton NE conditions and target mode selection T_MS_ versus other assistance modes. The limited discriminative power of α in distinguishing T_MS_ from other assistance modes underscores $\eta^{\prime }s$ enhanced capability for detailed quantification of muscle synergy pattern similarity. This is crucial for precise classification of each assistive condition, and has significance implications for customized exoskeleton design and control of (Hassan et al., 2018).

From another perspective, our proposed synergy similarity index η directly quantifies similarity between synergy vectors through spatial comparison of muscle activation patterns, making it particularly sensitive to spatial changes in muscle coordination. In contrast, the referred index α indirectly assesses similarity by comparing sEMG data when synergy weights are substituted by the compared synergy weights, resulting in reduced sensitivity to magnitude differences. These differences in calculation principles may explain why η detected more significant differences in our study, as it directly captures spatial aspects of synergy patterns. The use of η may be particularly valuable in human-robot-interaction applications, as it provides a more direct and sensitive measure of how exoskeleton assistance alters muscle coordination, which is essential for optimizing assistive strategies. α may be computationally simpler, its reduced sensitivity to magnitude changes could limit its utility in designing personalized exoskeleton control systems.

Our findings also provide novel insights into neuromuscular adaptation processes during exoskeleton use. Figure 4 demonstrates that healthy subjects effectively adjust their muscle recruitment and coordination patterns in response tohip exoskeleton assistance, as evidenced by changes in muscle synergy similarity. This observation aligns with previous findings regarding adaptation to ankle exoskeleton (Steele et al., 2017). Notably, our study addresses a significant gap in the literature by systematically investigating the temporal dynamics of muscle synergy adaptation during transitions between different robotic assistance levels. This temporal analysis of adaptation processes represents an important contribution to the field of human-robot interaction and exoskeleton control optimization.

### 4.3 Synergy adaptation

The temporal analysis of muscle synergy similarityrevealed distinct adaptation patterns during transitions between different exoskeleton assistance modes (Figure 7). Notably, subjects could adjust their muscle recruitment and coordination within a remarkably short time (<2 min) when transitioning from ZT to T2 assistance mode. This adaptation process consistently exhibited two distinct phases: an initial similarity change (indicated by the black arrows), followed by stabilization to a relatively constant state. The same biphasic pattern was observed during subsequent transitions from T2 to T1 mode, suggesting a systematic neuromuscular adaptation mechanism involving coordinated adjustments in muscle recruitment patterns. These findings aligns with previous observations of muscle synergy modulation during exoskeleton use (T. Zhang et al., 2019), indicating that theneuromuscular system undergoes rapid reorganization in response to altered biomechanical demands.

While both similarity indices ( η and α ) captured this adaptation pattern, η demonstrated superior sensitivity, as evidenced by its more pronounced range of change compared to α . The direction and duration of α varied significantly across individuals, potentially reflecting inter-subject differences in optimal assistance requirements. One possible explanation is that T2 mode may not be the theoretically optimal assistance mode for everyone, which aligns with the results from Figure 6, Supplementary Tables SA1, A2. Subject one exhibited an initial increase in similarity during the adaptation process, followed by a gradual stabilization. This is consistent with the values of both similarity indices in the variable peak torque test, where they were greater in T2 mode than in T1 mode. Conversely, subject four showed a decline in similarity during the adaptation process, followed by a plateau, consistent with the similarity values in the variable peak torque test where T2 mode exhibited lower similarity than T1 mode. Additionally, except for subject six (poor quality of the sEMG acquisition), most subjects followed this pattern.

Notably, some subjects (e.g., Subject 8) showed minimal similarity changes during T2 condition, potentially attributable to two factors: (1) optimized torque patterns that closely matched natural gait dynamics, resulting in less disruptive assistance, or (2) relatively low torque amplitudes that minimally influenced muscle recruitment patterns. Although T2 mode provided subjectively comfortable assistance, our findings emphasize the importance of considering individual biomechanical responses when optimizing exoskeleton control strategies.

### 4.4 Study limitations

This study has several limitations that warrant consideration. First, the relatively small sample size (n = 8) may limit the generalizability of our findings.Additionally, the quality of sEMG data was not optimal during the whole tests for some participants. Future studies should incorporate a larger, more participant pool and implement rigorous quality control measures for sEMG data acquisition to enhance the robustness of the findings.

Second, only a limited amplitude range of assistive torque profile was applied to the hip exoskeleton for walking assistance. Changing the torque profile (including timing and amplitude for each feature point) may lead to more obvious variance in synergy similarity. However, without limited timing and amplitude for each feature point, it will take long time for subjects to adjust to the assistance, which may be even unsafe. Moreover, free walking trials should be conducted instead of treadmill walking with a constant speed since in real life, humans would constantly adjust their walking speed and perform more natural and relaxed musculoskeletal states.

## 5 Conclusion

This study investigated muscle synergies during treadmill walking with and without a portable hip exoskeleton, introducing a novel evaluation index for quantifying muscle synergy similarity across various assistive conditions. Our findings demonstrate that the human neuromuscular system exhibits remarkable adaptability in response to external robotic assistance, as evidenced by dynamic changes in synergy patterns. The proposed similarity index effectively captured these adaptive responses, revealing systematic adjustments in muscle recruitment and coordination strategies (Figure 7). These results contribute to our understanding of neuromuscular adaptation mechanisms during exoskeleton-assisted gait, addressing a critical gap in the field of wearable robotics. The observed adaptation patterns suggest that the human neuromuscular system rapidly reorganizes its control strategies to accommodate external assistance, highlighting the potential for developing more effective exoskeleton control paradigms.Future work should more extensively study the relationship between muscle synergy and other aspects like muscle activities, metabolic expenditure, and user subjective preference. Such investigations could inform the development of more natural and intuitive exoskeleton control frameworks, ultimately enhancing mobility assistance for both healthy individuals and those with neuromuscular disorders in real-world settings.

## Data Availability

The raw data supporting the conclusions of this article will be made available by the authors, without undue reservation.
