# Supplementary material for: Characterization of muscle synergy similarity and adaptation in hip exoskeleton-assisted locomotion
Source: Front Bioeng Biotechnol. 2025 Sep 19;13:1679101. doi: 10.3389/fbioe.2025.1679101 (PMC12491322; doi:10.3389/fbioe.2025.1679101)
Supplement: Supplementary file 1 [file Table1.docx]

# **Appendix**

**Table A1. Median values of two similarity indices for different participants under various assistance conditions.**

| **Patterns**  **and**  **indices** |  | **NE** | |  | **ZT** | |  | **T1** | |  |  | **T2** | |  | **T3** | |  | **T4** | |  | **T5** | |  | **T6** | |  | |
| --- | --- | --- | --- | --- | --- | --- | --- | --- | --- | --- | --- | --- | --- | --- | --- | --- | --- | --- | --- | --- | --- | --- | --- | --- | --- | --- | --- |
|  |  | $\boldsymbol{\eta}_{\boldsymbol{m}}$ | $\boldsymbol{\alpha}_{\boldsymbol{m}}$ |  | $\boldsymbol{\eta}_{\boldsymbol{m}}$ | $\boldsymbol{\alpha}_{\boldsymbol{m}}$ |  | $\boldsymbol{\eta}_{\boldsymbol{m}}$ | $\boldsymbol{\alpha}_{\boldsymbol{m}}$ |  |  | $\boldsymbol{\eta}_{\boldsymbol{m}}$ | $\boldsymbol{\alpha}_{\boldsymbol{m}}$ |  | $\boldsymbol{\eta}_{\boldsymbol{m}}$ | $\boldsymbol{\alpha}_{\boldsymbol{m}}$ |  | $\boldsymbol{\eta}_{\boldsymbol{m}}$ | $\boldsymbol{\alpha}_{\boldsymbol{m}}$ |  | $\boldsymbol{\eta}_{\boldsymbol{m}}$ | $\boldsymbol{\alpha}_{\boldsymbol{m}}$ |  | $\boldsymbol{\eta}_{\boldsymbol{m}}$ | $\boldsymbol{\alpha}_{\boldsymbol{m}}$ |  |  |
| **Subject 1** |  | 0.9467 | 0.8856 |  | 0.6792 | 0.8367 |  | 0.6683 | 0.8172 |  |  | 0.7070 | **0.8457** |  | **0.7548** | 0.8304 |  | 0.6860 | 0.8380 |  | **—** | **—** |  | **—** | **—** |  |  |
| **Subject 2** |  | 0.7995 | 0.6774 |  | 0.5494 | 0.6196 |  | 0.6623 | 0.6539 |  |  | 0.5765 | 0.6491 |  | **0.7336** | **0.7075** |  | 0.7195 | 0.6602 |  | **—** | **—** |  | **—** | **—** |  |  |
| **Subject 3** |  | 0.9847 | 0.8703 |  | 0.6533 | 0.7040 |  | 0.6129 | 0.6975 |  |  | 0.7024 | 0.7229 |  | **0.7926** | **0.8048** |  | 0.7090 | 0.7734 |  | **—** | **—** |  | **—** | **—** |  |  |
| **Subject 4** |  | 0.8321 | 0.9807 |  | 0.7321 | 0.9500 |  | 0.7034 | 0.9309 |  |  | 0.6703 | 0.9326 |  | **0.7555** | **0.9387** |  | 0.6519 | 0.9228 |  | 0.6142 | 0.9032 |  | 0.5970 | 0.8878 |  |  |
| **Subject 5** |  | 0.7015 | 0.9427 |  | 0.6833 | 0.9488 |  | 0.7035 | 0.9398 |  |  | 0.7438 | 0.9339 |  | 0.7490 | 0.9329 |  | **0.7638** | **0.9505** |  | 0.7548 | 0.9273 |  | 0.7590 | 0.9288 |  |  |
| **Subject 6** |  | 0.8020 | 0.9591 |  | 0.5283 | 0.8814 |  | 0.3958 | 0.7871 |  |  | 0.3480 | 0.8145 |  | 0.4368 | 0.8430 |  | 0.4841 | 0.8204 |  | 0.4831 | 0.8434 |  | **0.5438** | **0.8648** |  |  |
| **Subject 7** |  | 0.8773 | 0.9205 |  | 0.7540 | 0.8366 |  | 0.8402 | **0.8603** |  |  | 0.8515 | 0.8564 |  | 0.8714 | 0.8479 |  | **0.8826** | 0.8332 |  | 0.8809 | 0.8412 |  | 0.8538 | 0.8366 |  |  |
| **Subject 8** |  | 0.7477 | 0.9454 |  | 0.6068 | 0.8545 |  | 0.6727 | 0.8939 |  |  | 0.7696 | **0.9099** |  | **0.7763** | 0.8986 |  | 0.6812 | 0.8616 |  | 0.6200 | 0.8577 |  | 0.5305 | 0.7579 |  |  |

*Note*. Eight participants underwent testing across diverse assistance modes, yielding median values for two collaborative similarity indices, $\eta_{m}$ and $\alpha_{m}$, within each experimental group. Bold figures in each row signify the two indices (maximum medians) corresponding to the assistance mode exhibiting the highest individual synergy similarity.
