# Supplementary material for: Characterization of muscle synergy similarity and adaptation in hip exoskeleton-assisted locomotion
Source: Front Bioeng Biotechnol. 2025 Sep 19;13:1679101. doi: 10.3389/fbioe.2025.1679101 (PMC12491322; doi:10.3389/fbioe.2025.1679101)
Supplement: Supplementary file 2 [file Table2.docx]

**Table A2. Significant differences in two synergy similarity indices *η* and *α* under various assistance modes.**

| **Patterns** | **Subject 1** | | | | **Subject 2** | | | | **Subject 3** | | | | | **Subject 4** | | | |
| --- | --- | --- | --- | --- | --- | --- | --- | --- | --- | --- | --- | --- | --- | --- | --- | --- | --- |
| **and** | $\eta$ | | $\alpha$ | | $\eta$ | | $\alpha$ | | $\eta$ | | $\alpha$ | | | $\eta$ | | $\alpha$ | |
| **indices** | **95%CI** | **P** | **95%CI** | **P** | **95%CI** | **P** | **95%CI** | **P** | **95%CI** | **P** | **95%CI** | **P** | **95%CI** | | **P** | **95%CI** | **P** |
| **NE&ZT** | 0.2068~0.2600 | <0.0001 | -0.0112~0.0419 | 0.5638 | 0.2162~0.274 | <0.0001 | 0.0213~0.0792 | <0.0001 | 0.2161~0.2895 | <0.0001 | 0.0865~0.1630 | <0.0001 | 0.0444~0.1165 | | <0.0001 | 0.0004~0.0725 | 0.045 |
| **NE&T1** | 0.2601~0.3131 | <0.0001 | 0.0238~0.0769 | <0.0001 | 0.1245~0.1823 | <0.0001 | 0~0.0578 | 0.0502 | 0.2758~0.3492 | <0.0001 | 0.1090~0.1855 | <0.0001 | 0.0909~0.1630 | | <0.0001 | 0.0129~0.0849 | 0.0011 |
| **NE&T2** | 0.1972~0.2502 | <0.0001 | -0.0200~0.0330 | 0.9821 | 0.1919~0.2497 | <0.0001 | 0.0024~0.0603 | 0.0246 | 0.1981~0.2714 | <0.0001 | 0.0941~0.1705 | <0.0001 | 0.1321~0.2041 | | <0.0001 | 0.0174~0.0895 | 0.0002 |
| **NE&T3** | 0.1712~0.2242 | <0.0001 | 0.0012~0.0542 | 0.035 | 0.0331~0.0909 | <0.0001 | -0.0541~0.0037 | 0.1281 | 0.1147~0.1880 | <0.0001 | 0.0094~0.0859 | 0.0053 | 0.0558~0.1279 | | <0.0001 | 0.0061~0.0782 | 0.0096 |
| **NE&T4** | 0.2094~0.2624 | <0.0001 | -0.0149~0.0381 | 0.8137 | 0.0609~0.1187 | <0.0001 | -0.0055~0.0523 | 0.1905 | 0.2095~0.2829 | <0.0001 | 0.0414~0.1178 | <0.0001 | 0.1367~0.2087 | | <0.0001 | 0.0209~0.0930 | <0.0001 |
| **NE&T5** | / | / | / | / | / | / | / | / | / | / | / | / | 0.1571~0.2292 | | <0.0001 | 0.0508~0.1229 | <0.0001 |
| **NE&T6** | / | / | / | / | / | / | / | / | / | / | / | / | 0.1929~0.265 | | <0.0001 | 0.0596~0.1316 | <0.0001 |
| **T_MS_&ZT** | -0.0623~-0.0091 | 0.0019 | -0.0354~0.0177 | 0.9322 | -0.2121~-0.1542 | <0.0001 | -0.1043~-0.0465 | <0.0001 | -0.1381~-0.0648 | <0.0001 | -0.1154~-0.0389 | <0.0001 | -0.0247~0.0474 | | 0.9798 | -0.0304~0.0417 | 0.9998 |
| **T_MS_&T1** | -0.1154~-0.0624 | <0.0001 | -0.0704~-0.0173 | <0.0001 | -0.1203~-0.0625 | <0.0001 | -0.0830~-0.0252 | <0.0001 | -0.1978~-0.1245 | <0.0001 | -0.1379~-0.0614 | <0.0001 | -0.0711~0.001 | | 0.0628 | -0.0428~0.0292 | 0.9992 |
| **T_MS_&T2** | -0.0525~0.0006 | 0.0589 | — | — | -0.1877~-0.1299 | <0.0001 | -0.0855~-0.0276 | <0.0001 | -0.1201~-0.0468 | <0.0001 | -0.1229~-0.0464 | <0.0001 | -0.1123~-0.0402 | | <0.0001 | -0.0474~0.0247 | 0.9803 |
| **T_MS_&T3** | — | — | -0.0053~0.0477 | 0.2025 | — | — | — | — | — | — | — | — | — | | — | — | — |
| **T_MS_&T4** | 0.0117~0.0647 | 0.0006 | -0.0214~0.0316 | 0.5092 | -0.0011~0.0568 | 0.0664 | 0.0197~0.0775 | <0.0001 | 0.0582~0.1315 | <0.0001 | -0.0063~0.0702 | 0.1616 | 0.0448~0.1169 | | <0.0001 | -0.0212~0.0509 | 0.9165 |
| **T_MS_&T5** | / | / | / | / | / | / | / | / | / | / | / | / | 0.0653~0.1373 | | <0.0001 | 0.0087~0.0807 | 0.0043 |
| **T_MS_&T6** | / | / | / | / | / | / | / | / | / | / | / | / | 0.1011~0.1731 | | <0.0001 | 0.0175~0.0895 | 0.0002 |
| **Patterns and indices** | **Subject 5** | | | | **Subject 6** | | | | **Subject 7** | | | | **Subject 8** | | | | |
|  | $\eta$ | | $\alpha$ | | $\eta$ | | $\alpha$ | | $\eta$ | | $\alpha$ | | $\eta$ | | | $\alpha$ | |
|  | **95%CI** | **P** | **95%CI** | **P** | **95%CI** | **P** | **95%CI** | **P** | **95%CI** | **P** | **95%CI** | **P** | **95%CI** | | **P** | **95%CI** | **P** |
| **NE&ZT** | -0.0230~0.0462 | 0.9719 | -0.0392~0.0300 | >0.9999 | 0.1734~0.2688 | <0.0001 | 0.0423~0.1377 | <0.0001 | 0.0681~0.1414 | <0.0001 | 0.0524~0.1257 | <0.0001 | 0.0902~0.1811 | | <0.0001 | 0.0473~0.1383 | <0.0001 |
| **NE&T1** | -0.0371~0.0320 | >0.9999 | -0.0293~0.0399 | 0.9998 | 0.2979~0.3933 | <0.0001 | 0.1232~0.2186 | <0.0001 | -0.0176~0.0557 | 0.7621 | 0.0198~0.0930 | <0.0001 | 0.0221~0.113 | | 0.0002 | 0.0045~0.0955 | 0.0196 |
| **NE&T2** | -0.0637~0.0054 | 0.1722 | -0.0256~0.0436 | 0.9936 | 0.3584~0.4538 | <0.0001 | 0.0911~0.1865 | <0.0001 | -0.0026~0.0707 | 0.0903 | 0.0310~0.1042 | <0.0001 | -0.0470~0.044 | | >0.9999 | -0.0124~0.0786 | 0.3458 |
| **NE&T3** | -0.0728~-0.0037 | 0.0184 | -0.0196~0.0496 | 0.893 | 0.2730~0.3684 | <0.0001 | 0.0716~0.1671 | <0.0001 | -0.0632~0.0100 | 0.3487 | 0.0328~0.1061 | <0.0001 | -0.0605~0.0305 | | 0.9744 | -0.0071~0.0839 | 0.1699 |
| **NE&T4** | -0.0898~-0.0207 | <0.0001 | -0.0375~0.0317 | >0.9999 | 0.2329~0.3284 | <0.0001 | 0.0847~0.1801 | <0.0001 | -0.0746~-0.0014 | 0.0358 | 0.0534~0.1267 | <0.0001 | 0.0362~0.1272 | | <0.0001 | 0.0325~0.1235 | <0.0001 |
| **NE&T5** | -0.0913~-0.0221 | <0.0001 | -0.0166~0.0525 | 0.7636 | 0.2191~0.3146 | <0.0001 | 0.0746~0.1700 | <0.0001 | -0.0676~0.0057 | 0.1703 | 0.0410~0.1143 | <0.0001 | 0.0957~0.1866 | | <0.0001 | 0.0362~0.1271 | <0.0001 |
| **NE&T6** | -0.0891~-0.0200 | <0.0001 | -0.0161~0.0530 | 0.7379 | 0.1674~0.2629 | <0.0001 | 0.0450~0.1405 | <0.0001 | -0.0434~0.0299 | 0.9993 | 0.0499~0.1232 | <0.0001 | 0.1675~0.2585 | | <0.0001 | 0.1350~0.2260 | <0.0001 |
| **T_MS_&ZT** | -0.1014~-0.0322 | <0.0001 | -0.0329~0.0363 | >0.9999 | -0.0537~0.0418 | >0.9999 | -0.0450~0.0505 | >0.9999 | -0.1794~-0.1061 | <0.0001 | -0.0693~0.0040 | 0.1218 | -0.1961~-0.1051 | | <0.0001 | -0.1052~-0.0142 | 0.0019 |
| **T_MS_&T1** | -0.0873~-0.0181 | 0.0001 | -0.0428~0.0264 | 0.9963 | -0.1781~-0.0827 | <0.0001 | -0.1259~-0.0305 | <0.0001 | -0.0937~-0.0204 | <0.0001 | — | — | -0.1280~-0.0370 | | <0.0001 | -0.0624~0.0286 | 0.9502 |
| **T_MS_&T2** | -0.0607~0.0085 | 0.2977 | -0.0465~0.0227 | 0.9668 | -0.2387~-0.1432 | <0.0001 | -0.0938~0.0017 | 0.0674 | -0.1087~-0.0354 | <0.0001 | -0.0255~0.0478 | 0.9834 | -0.0589~0.0321 | | 0.9863 | — | — |
| **T_MS_&T3** | -0.0516~0.0176 | 0.8108 | -0.0525~0.0167 | 0.767 | -0.1532~-0.0578 | <0.0001 | -0.0743~0.0211 | 0.6906 | -0.0480~0.0252 | 0.9815 | -0.0236~0.0497 | 0.96 | — | | — | -0.0402~0.0508 | >0.9999 |
| **T_MS_&T4** | — | — | — | — | -0.1132~-0.0178 | 0.0009 | -0.0874~0.0080 | 0.1858 | — | — | -0.0030~0.0703 | 0.0985 | 0.0512~0.1422 | | <0.0001 | -0.0006~0.0904 | 0.0557 |
| **T_MS_&T5** | -0.0360~0.0332 | >0.9999 | -0.0137~0.0555 | 0.5968 | -0.0994~-0.0040 | 0.023 | -0.0773~0.0181 | 0.5628 | -0.0296~0.0437 | 0.999 | -0.0154~0.0579 | 0.6466 | 0.1106~0.2016 | | <0.0001 | 0.0031~0.0940 | 0.0269 |
| **T_MS_&T6** | -0.0339~0.0353 | >0.9999 | -0.0132~0.0559 | 0.5673 | — | — | — | — | -0.0054~0.0679 | 0.1603 | -0.0065~0.0668 | 0.1955 | 0.1825~0.2735 | | <0.0001 | 0.1019~0.1929 | <0.0001 |

*Note.* Eight participants underwent a significance analysis to assess disparities in two synergy similarity indices across diverse assistance modes. This involved comparing these indices between scenarios of NE and wearing exoskeletons, as well as between the assistance mode displaying the highest individual synergy similarity (T_MS_) and alternative assistance modes. The T_MS_ aligns with the individuals' highest $\eta_{m}$ and $\alpha_{m}$ values, as demonstrated in Table A1. In this table, 95%CI stands for 95% confidence intervals for all effect size estimates.
